# Cerina: systematic circRNA functional annotation based on integrative analysis of ceRNA interactions

**DOI:** 10.1038/s41598-020-78469-x

**Published:** 2020-12-17

**Authors:** Jacob Cardenas, Uthra Balaji, Jinghua Gu

**Affiliations:** Baylor Scott & White Research Institute, Dallas, TX 75204 USA

**Keywords:** Data integration, Databases

## Abstract

Circular RNAs, a family of covalently circularized RNAs with tissue-specific expression, were recently demonstrated to play important roles in mammalian biology. Regardless of extensive research to predict, quantify, and annotate circRNAs, our understanding of their functions is still in its infancy. In this study, we developed a novel computational tool: Competing Endogenous RNA for INtegrative Annotations (Cerina), to predict biological functions of circRNAs based on the competing endogenous RNA model. Pareto Frontier Analysis was employed to integrate ENCODE mRNA/miRNA data with predicted microRNA response elements to prioritize tissue-specific ceRNA interactions. Using data from several circRNA-disease databases, we demonstrated that Cerina significantly improved the functional relevance of the prioritized ceRNA interactions by several folds, in terms of precision and recall. Proof-of-concept studies on human cancers and cardiovascular diseases further showcased the efficacy of Cerina on predicting potential circRNA functions in human diseases.

## Introduction

Circular RNAs (circRNAs) are a family of RNAs that form circular structures by joining the 3′ and 5′ ends covalently. Although originally considered as by-products of “splicing noise”^1,2^, researchers have recently discovered that circRNAs play important roles in human diseases, including cancers, neurological diseases, heart and vascular diseases, among many others. CircRNAs are highly stable and display tissue-specific expression patterns, making them promising candidates as disease biomarkers^3–7^.

Despite the rapid growth in cataloging new circRNAs, their biological functions in human diseases are yet largely unknown. Among many putative mechanisms, such as interaction with RNA binding proteins (RBP), alternative splicing competition, posttranscriptional gene regulation, and protein coding, one of the most well-studied circRNA functions is to act as the competing endogenous RNA (ceRNA) or miRNA “sponge”^3,7–13^. In the ceRNA model^14,15^, linear RNAs and circRNAs competitively interact with miRNAs through miRNA response elements (MREs) to leverage the amount of active miRNAs in a cell, which has been extensively demonstrated in a variety of diseases^6,16,17^. One notable ceRNA examples is CDR1as, a brain-enriched circRNA that is found to function as miRNA sponge for miR-7^18,19^ in various human diseases, including colon cancer^20^, gastric cancer^21^, esophageal cancer^22^, and myocardial infarction^23^. A number of other ceRNA interactions have also been uncovered in cancer, such as circPVT1-miR125 in gastric cancer^24^, circITCH-miR7/miR214 in lung cancer^25^, circHIPK3-miR124 in liver cancer^26^, and circTTBK2-miR217 in glioma^27^. Other than cancer, the role of circRNA as microRNA sponge is also under heavy investigation in cardiovascular diseases^28,29^. In addition to beforementioned CDR1as-miR7 interaction in myocardial infarction, new theories have emerged hypothesizing the involvement of various circRNAs in multiple cardiovascular diseases through sequestration of miRNAs, exampled by CircRNA_081881-miR548^30^ and MFCAR-miR652^31^ interactions in ischemia/reperfusion injury and myocardial infarction, sponging effect and therapeutic potential of circRNA_000203 and cirRNA_010567 in cardiac fibrosis^32,33^, and protective effect of circRNA HRCR against hypotrophy and heart failure by sequestering miR223^34^. It was also reported that regulation of disordered vascular smooth muscle cell proliferation and migration through the circWDR77-miR124-FGF2 axis was vital in atherosclerosis pathogenesis^35^. In neurological disease research, in addition to the prominent role of CDR1as-miR7 sponging events in Alzheimer’s disease^36,37^, hundreds of circRNA were recently identified from multiple high-throughput studies to investigate circRNA-miRNA-mRNA interactions related to AD pathogenesis^38,39^.

Recently, there has been a surge of interest in annotating circRNA functions. Databases such as CircInteractome^40^, CircAtlas^41^, Circ2Traits^42^, CircNet^43^, TSCD^44^, CSCD^45^, and Circbank^46^, among others, have collected predicted MRE sites to bridge individual circRNAs to their potential functions through miRNAs. Some of these resources also report circRNA-interacting RBPs, due to their roles in circRNA formation, translation, targeted gene regulation, and transport^47^. Additionally, Circ2Traits annotated circRNAs that harbored disease-related single nucleotide polymorphisms (SNPs) as putative evidence for circRNA-disease associations^42^. CircRNADb compiled detailed information regarding internal ribosomal entry site (IRES) and open reading frame (ORF) to implicate possible protein-coding potential of circRNAs^48^. Recently, CircFunBase has reported a collection of more than 7,000 manually curated circRNAs on 15 different species, which is among the first to systematically summarize circRNA functions based on circRNA differential expression data^49^.

Amid the existing efforts to annotate individual circRNAs and their potential functions, tools for systematic circRNA functional annotation and pathway analysis are still lacking. In this work, we developed a novel computational tool for circRNA functional analysis: Competing Endogenous RNA for INtegrative Annotations (Cerina). As the first statistical method for systematic circRNA functional analysis, Cerina has several major technical advances. Firstly, Cerina paired up circRNA, linear RNA, and miRNA expression data for 11 human organs from ENCODE^50^ and jointly analyzed them for the first time, allowing comprehensive pan-tissue profiling of ceRNA expressions. Secondly, expression and binding data for ceRNAs are integrated and prioritized based on the principles of Pareto optimality, which further increased the biological relevance of predicted ceRNA interactions. Finally, a user-friendly, web-based interface is made available for users to query a circRNA and retrieve its interacting miRNAs, their significant target genes, and the enriched biological functions and pathways.

## Methods

### Processing of sequencing data

29 total RNA-Seq samples and 39 miRNA-Seq samples from 11 ENCODE tissues were analyzed in this study (Supplementary Table S1). Fastq files from replicate samples were merged before downstream analysis.

#### ENCODE total RNA-Seq data (linear RNA)

Sequencing quality control of ENCODE total RNA-Seq data were performed by FastQC (https://www.bioinformatics.babraham.ac.uk/projects/fastqc/). Adapter sequences were trimmed and low-quality reads (< 20) were filtered using cutadapt^51^. Reads from total RNA sequencing were aligned to human genome (hg19/GRCh37) using hisat2^52^ and converted to BAM format using samtools^53^. The featureCounts^54^ tool was used to assign reads to each gene in the GENCODE^55^ GTF file (https://www.gencodegenes.org/human/release_19.html). Counts from the total RNA-Seq pipeline were used to approximate linear RNA gene expression. After filtering out low-expressing genes (total read counts < 10), DESeq2^56^ was used for count data normalization. A final layer of filtering was applied by removing genes with mean normalized counts less than 10. Counts per million (CPM) is used to report and visualize mRNA expression levels.

#### ENCODE total RNA-Seq data (circular RNA)

We used back-splicing (BS) junction reads to approximate circRNA gene expression from ENCODE total RNA-Seq data. It was previously reported that individual circRNA detection method suffered from high false positives in BS junction prediction, yet combining results from two different algorithms (i.e., intersection) is a simple and effective remedy to significantly reduce false positives^57^. In a recent review paper^58^ comparing a dozen circRNA detection algorithms, CIRI2^59,60^ and CIRCexplorer^61^ demonstrated their best overall performance in terms of accuracy and efficiency based on the simulated and real RNaseR + data sets. Hence, we developed a framework that combines predictions from both CIRI2 and CIRCexplorer to improve the specificity of circRNA detection (Supplementary Methods). Spliced reads per billion mapped reads (SRPBM) is used to report and visualize circRNA expression levels.

#### ENCODE miRNA-Seq

The extra-cellular RNA processing toolkit (exceRpt)^62^ was used to process ENCODE miRNA sequencing data. Firstly, exceRpt filtered reads were mapped to UniVec vectors and ribosomal RNA sequences, followed by alignment of the remaining reads to the human genome (hg19) and then quantified for different types of RNAs, including miRNAs. The same normalization and filtering criteria were applied to process ENCODE miRNA data. CPM is used to report and visualize miRNA expression levels.

### Prediction of miRNA binding sites on linear/circular RNA

Among a total of 33,461 confidently detected circRNA from ENCODE total RNA-Seq data, 30,282 had mature sequences (hg19) from circAtlas^41^ available for download at http://159.226.67.237/zhao/Data/circAtlas_supply/human_sequence_v1909.txt.zip. Mature sequences of novel circRNAs were estimated using a hierarchical framework described in the Supplementary Methods.

Perl script from TargetScan 7.1^63^ was used to identify MREs based on circRNA mature sequences. For each circRNA, the number of MREs was normalized by the length of its mature splice sequence and defined as the circRNA-miRNA binding scores ${S}_{\text{MRE}}^{\text{circ}|\text{mir}}$.

miRNA-gene (i.e., linear RNA) binding data were download from TargetScan 7.2 (http://www.targetscan.org/vert_72/vert_72_data_download/Conserved_Family_Info.txt.zip, http://www.targetscan.org/vert_72/vert_72_data_download/Nonconserved_Family_Info.txt.zip) and used to form miRNA-gene scores, ${S}_{\text{MRE}}^{\text{mir}|\text{gene}}$. In addition to TargetScan, miRTarBase 7.0^64^, a curated database of experimentally validated miRNA-target gene interactions (${S}_{\text{MTB}}^{\text{mir}|\text{gene}}$) was used as another component of evidence. For each miRNA-gene pair, the number of MREs from TargetScan and the number of publications from miRTarBase were integrated to obtain the final miRNA-gene binding score (${S}_{P}^{\text{mir}|\text{gene}}$) based on the Pareto Frontier Analysis described below.

### Pan-tissue co-expression analysis

Most existing databases predict circRNA-miRNA interactions solely relying on sequence-based algorithms, which completely ignore tissue-specific expression information of circRNAs^41,44,65^. In order to form effective ceRNA networks, circRNA and miRNA both need to be expressed in the same tissue. Memczak et al. reported that CDR1as and miR-7 were both highly expressed in brain tissues, but not necessarily in other non-neuronal tissues^18^, which renders CDR1as a hallmark miR-7 sponge in neuronal tissues. Moreover, Guo et al. further argued that functional miRNA sponges require circRNAs to be expressed at consequential levels in the cell^66^. Therefore, incorporating circRNA and miRNA expression into ceRNA network analysis can help filter out false positive interactions with low or no expression. To this end, we assigned circRNA-miRNA expression scores ${S}_{\text{exp}}^{\text{circ}|\text{mir}}$ to all interactions by the following methodology. First, for each tissue, circRNAs/miRNAs with no expression are excluded and the ones passed filtering were utilized to calculate the empirical cumulative distribution function (ECDF) on the mean normalized expression, giving rise to tissue-specific circRNA and miRNA ECDF scores, ${S}_{\text{exp}}^{\text{circ}|\text{tissue}}$ and ${S}_{\text{exp}}^{\text{mir}|\text{tissue}}$, that take values on (0, 1]. We then defined circRNA-miRNA score as ${S}_{\text{exp}}^{\text{circ}|\text{mir}|\text{t}}={\text{min}}_{t\in T}\left\{{S}_{\text{exp}}^{\text{circ}|\text{t}},{S}_{\text{exp}}^{\text{mir}|\text{t}}\right\}$, and the final circRNA-miRNA score across all tissues is given by ${S}_{\text{exp}}^{\text{circ}|\text{mir}}={\text{max}}_{t\in T}{S}_{\text{exp}}^{\text{circ}|\text{mir}|\text{t}}$, where *T* is the set of all tissues from ENCODE. Assigning scores in this manner ensures that a high ${S}_{\text{exp}}^{\text{circ}|\text{mir}}$ coincides with relatively high expression of the circRNA and miRNA in at least one tissue.

### Integrative analysis of ceRNA interaction data using Pareto Frontier analysis

In order to improve the quality and functional relevance of the predicted ceRNA interactions, we integrated miRNA binding data and gene expression data using Pareto Frontier analysis (PFA). PFA is a technology to resolve the challenge of balancing among multiple competing objectives simultaneously to achieve an overall ranking optimality. In our case, it means to derive a combined interaction score between miRNAs and linear/circular RNAs based on various data types, such as expression data and predicted miRNA binding data. A key concept in PFA is the Pareto dominance. In the context of ranking 2-dimensional miRNA-circRNA interaction scores (expression score ${f}_{1}$ and binding score ${f}_{2}$), Pareto dominance is defined as: given two pairs of interactions ${x}_{1}$ and ${x}_{2}$, ${x}_{2}$ is said to Pareto dominant ${x}_{1}$ if

$${f}_{n}\left({x}_{1}\right)\le {f}_{n}\left({x}_{2}\right), \quad \text{for all }\,n,$$

$${\text{and }f}_{n}\left({x}_{1}\right)<{f}_{n}\left({x}_{2}\right), \quad \text{for at least one }\,n,\,\text{ where }\,n\in \left\{\text{1,2}\right\}.$$

The principle of Pareto dominance can be easily generalized for combing more than two scores. It is particularly suitable for combining asymmetric information without directly making comparisons across different data types, nor subjectively choosing a trade-off between different competing objectives. For circRNA-miRNA interactions, the length-normalized circRNA-miRNA binding score ${S}_{\text{MRE}}^{\text{circ}|\text{mir}}$ and circRNA-miRNA co-expression score ${S}_{\text{exp}}^{\text{circ}|\text{mir}}$ were combined using the PFA method to re-rank all pairs of circRNA-miRNA interactions. The new rank of each interaction pair was re-scaled by the total number of interaction pairs to obtain a final combined interaction score ${S}_{P}^{\text{circ}|\text{mir}}$ between 0 and 1, where 1 denotes the strongest interaction and 0 denotes no evidence for a given interaction. The new combined score ${S}_{P}^{\text{circ}|\text{mir}}$ is also referred to as the Pareto score of the circRNA-miRNA interaction. For circRNA-miRNA interactions that fall on the same Pareto front, their Pareto scores will be the same. Similarly, for a miRNA-gene interaction, previously described scores ${S}_{\text{MRE}}^{\text{mir}|\text{gene}}$ and ${S}_{\text{MTB}}^{\text{mir}|\text{gene}}$ were also combined using the Pareto Frontier method to calculate a new Pareto score ${S}_{P}^{\text{mir}|\text{gene}}$. More details regarding PFA are provided in the Supplementary Methods.

### CircRNA functional enrichment analysis

Assigning functional annotations to an individual circRNA is based on the circRNA-miRNA-gene interaction framework we have built thus far, which consists of two steps: obtaining a list of significant genes and then testing functional enrichment of these genes. In the first step, given an individual circRNA $c$ and a set of $k$ miRNAs ${{\varvec{M}}}_{k}=\left[{m}_{1},{\dots ,m}_{k}\right]\subseteq {\varvec{M}}$, where ${S}_{P}^{c|{m}_{i}}>0$ for all $i\le k$ and ${\varvec{M}}$ is the full set of miRNAs. We define the circRNA-miRNA Pareto score vector as ${S}_{P}^{c|{{\varvec{M}}}_{k}}=\left[{S}_{P}^{c|{m}_{1}},{S}_{P}^{c|{m}_{2}},\dots ,{S}_{P}^{c|{m}_{k}}\right]$ and the miRNA-gene Pareto score matrix ($n\times k$).

$${S}_{P}^{{{\varvec{M}}}_{k}|{\varvec{G}}}\left[\left(\begin{array}{ccc}{S}_{P}^{{m}_{1}|{g}_{1}}& \cdots & {S}_{P}^{{m}_{k}|{g}_{1}}\\ \vdots & \ddots & \vdots \\ {S}_{P}^{{m}_{1}|{g}_{n}}& \cdots & {S}_{P}^{{m}_{k}|{g}_{n}}\end{array}\right)\right],$$

where ${\varvec{G}}$ is the set of genes $\left[{g}_{1},{\dots ,g}_{n}\right]$. We define the circRNA-gene Pareto score vector ${S}_{P}^{c|{\varvec{G}}}={S}_{P}^{c|{{\varvec{M}}}_{k}}\times {\left({S}_{P}^{{{\varvec{M}}}_{k}|{\varvec{G}}}\right)}^{\text{T}}=\left[{S}_{P}^{c|{g}_{1}},\dots ,{S}_{P}^{c|{g}_{n}}\right]$ as the final statistic to measure the predicted association between a given circRNA and the set of all genes. Following a similar procedure as Bleazard et al. to adjust for observed bias in miRNA functional enrichment analysis, we approximate the null distribution of ${S}_{P}^{c|{\varvec{G}}}$ (${S}_{P,\text{null},n}^{c|{\varvec{G}}}$) by randomly drawing $N$ (e.g., $N=\text{10,000}$) ${S}_{P}^{{{\varvec{M}}}_{k}|{\varvec{G}}}$ matrices from ${S}_{P}^{{\varvec{M}}|{\varvec{G}}}$ and re-computing ${S}_{P}^{c|{\varvec{G}}}$ under each iteration^67^, where $1\le n\le N$. Then, the statistical significance of a gene $g$ is given by p-value $=\frac{{\sum }_{n=1}^{N}\text{I}\left({S}_{P,\text{null},n}^{c|g}\ge {S}_{P}^{c|g}\right)+1}{N+1}$^68^, where $\text{I}(x)$ is the indicator function that equals 1 when $x$ is true, and 0 otherwise. Given a pre-specified statistical threshold (e.g., p-value ≤ 0.05), a list of genes surviving the threshold is identified. The second step, functional enrichment of significant genes, proceeds in standard fashion: apply Fisher’s exact test to test for overrepresentation on sets of functional terms. All gene sets, including KEGG^69–72^ pathways and gene ontology (GO)^72–74^ terms were obtained from the R package pathfindR^72^.

### Tools and software used

The Cerina tool is developed based on R^75^ and R shiny^76^, which also depends on several R packages (shinydashboard^77^, shinyjs^78^, shinycssloaders^79^, shinyBS^80^, DT^81^, tidyverse^82^, dendextend^83^, visNetwork^84^, heatmaply^85^, Matrix^86^, fastcluster^87^, htmltools^88^, reshape2^89^, and igraph^90^).

Additional R packages (ggolot2^91^, plotrix^92^, circlize^93^) were used to produce the figures in this paper. Cytoscape^94^ was used to create circRNA-miRNA-gene-function network for the prostate cancer case study. Commercial software Lucidchart (www.lucidchart.com) was used to assemble all final version of figures. All organ icons used in this paper were under Creative Commons liscence (CC BY 3.0, https://creativecommons.org/licenses/by/3.0/), which were obtained from iconfiner (https://www.iconfinder.com/) through Lucidchart without any changes. Images “Anatomy, blood, coronary, heart, organ icon” (https://www.iconfinder.com/icons/4312967/anatomy_blood_coronary_heart_organ_icon), “Anatomy, bowel, digestion, intestine, small icon” (https://www.iconfinder.com/icons/4312981/anatomy_bowel_digestion_intestine_small_icon), “Abdomen, anatomy, cavity, diaphragm, organ icon” (https://www.iconfinder.com/icons/4312964/abdomen_anatomy_cavity_diaphragm_organ_icon), “Abdomen, digestion, gaster, organ, stomach icon” (https://www.iconfinder.com/icons/4312980/abdomen_digestion_gaster_organ_stomach_icon), and “Abdomen, anatomy, liver, metabolism, organ icon” (https://www.iconfinder.com/icons/4312973/abdomen_anatomy_liver_metabolism_organ_icon) by Eucalyp Studio; “Organs, uterus icon” (https://www.iconfinder.com/icons/1609656/organs_uterus_icon) by Design Sciences. 

## Results

### Cerina overview: an integrative framework

**Figure 1:**
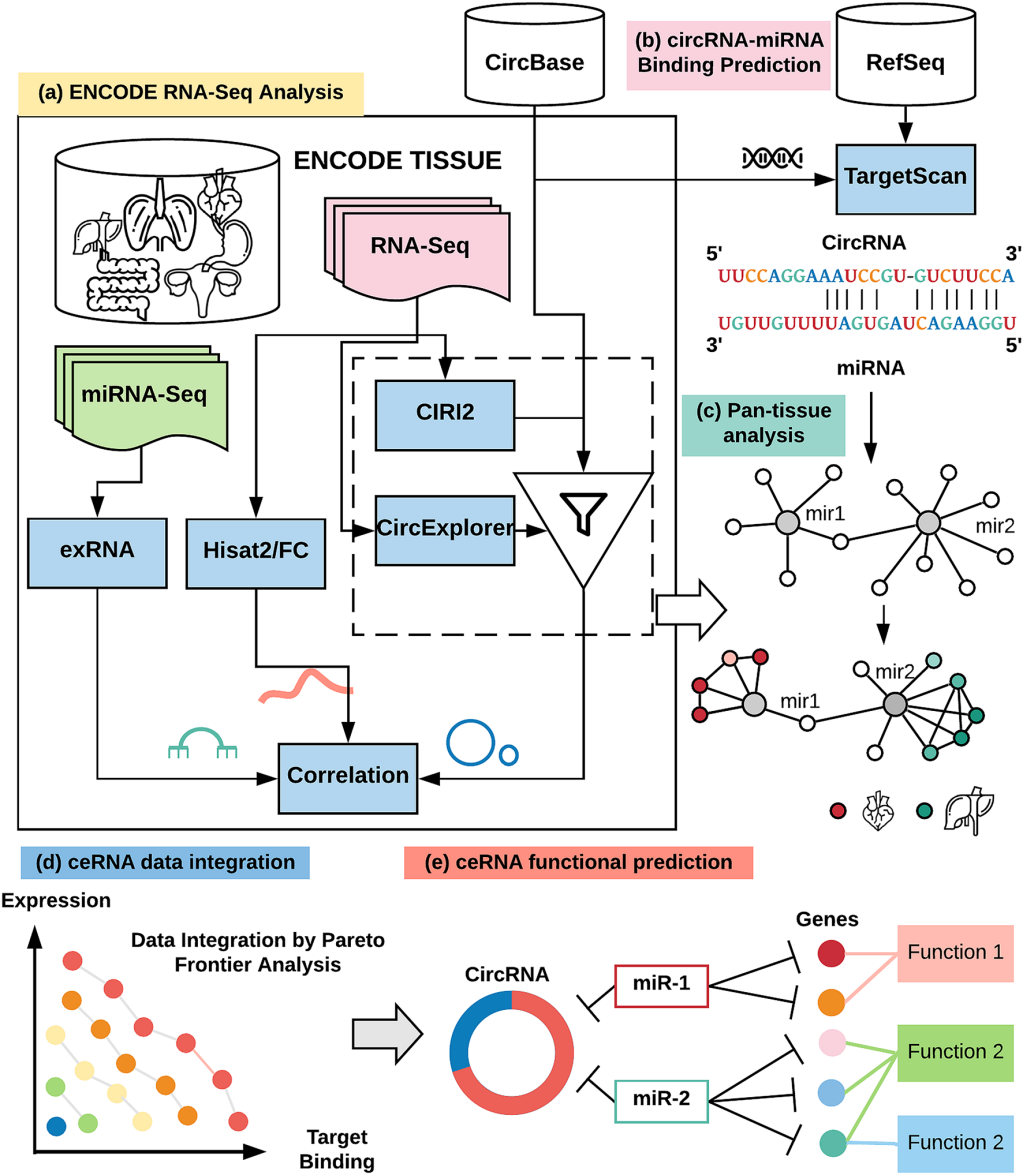
Flow chart of Cerina. (**a**) Analysis workflow for ENCODE tissue RNA-Seq/miRNA-Seq data. (**b**) Prediction of circRNA-miRNA bindings using TargetScan. (**c**) Pan-tissue analysis of ceRNA interactions. Incorporation of ENCODE gene expression data allows construct of tissue-specific circRNA-miRNA interaction networks. (**d**) Integrative analysis of ceRNA interactions. Pareto frontiers were calculated by integrating co-expression data with TargetScan-predict MRE data. (**e**) Based on the circRNA-miRNA-mRNA (gene)-function axis, circRNA functional prediction was performed by permuting the connections between a given circRNA and its interacting miRNAs/mRNAs. See section “Tools and software used” for license and attribution regarding the organ images used in the figure.

Figure 1 gives the flow chart of Cerina, which consists of several streamlined modules: starting from linear/circRNA expression quantification, MRE prediction, ceRNA interaction integration, to circRNA functional annotation. Briefly, paired total RNA and miRNA sequencing data of 11 human organs from the ENCODE project were downloaded and processed to generate linear RNA (coding and non-coding), miRNA, and circular RNA expression profiles (Fig. 1a). To reduce false-positive circRNA predictions, we combined results from CIRI2 and CircExplorer, two methods that were previously validated to have the best overall performance on the simulated and real datasets. Based on the estimated mature sequences of circRNAs, TargetScan 7.2 was employed to predict putative MREs, which were further normalized by the length of each circRNA’s mature splice sequence (Fig. 1b). Meanwhile, pan-tissue circRNA expression data were incorporated and tissue-specific ceRNA networks were also constructed (Fig. 1c). Following the Pareto dominance principle, all ceRNA interactions were ranked and grouped into a sequence of non-intersect sets called Pareto frontiers. These frontiers re-stratified all ceRNA interactions, integrating evidence from both gene expression and miRNA bindings. CeRNA interactions that fall onto the first Pareto frontier represent the circRNA-miRNA interactions with the highest confidence, either based on expression data or binding data, or both (Fig. 1d). Such procedure integratively re-prioritized a total of 1,540,275 ceRNA interactions between 33,455 circRNAs and 606 miRNAs detected in 11 ENCODE tissues. Finally, systems analysis was performed based on Pareto-ranked ceRNA interactions to identify top miRNAs, significant miRNA target genes, and enriched biological functions and pathways (Fig. 1e).

### Pareto Frontier analysis improves accuracy and functional relevance of ceRNA interactions

**Figure 2:**
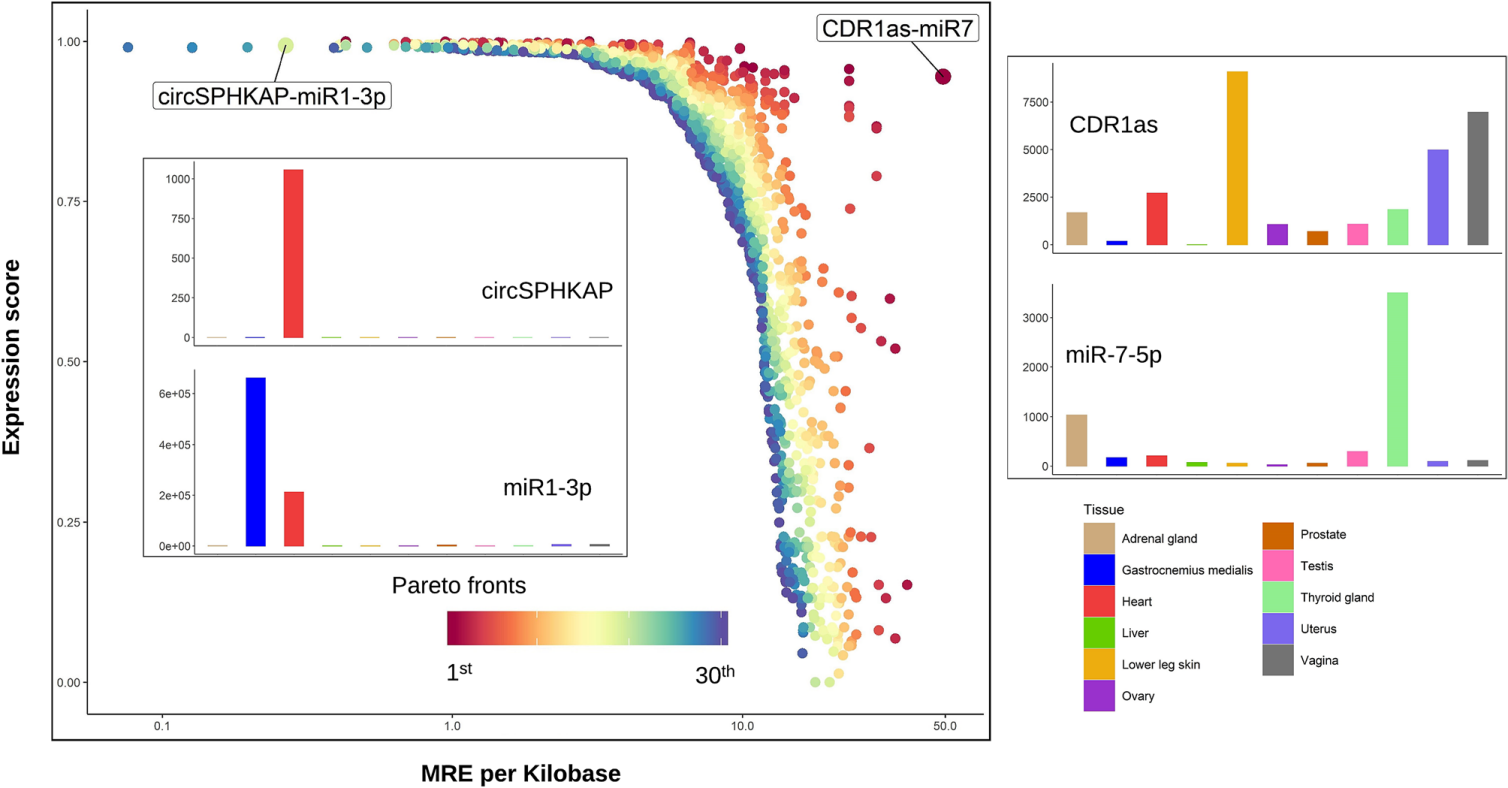
Pareto Frontier Analysis re-prioritize ceRNA interactions by integrating gene expression with MRE data. 2531 circRNA-miRNA interactions on the top 30 Pareto fronts. Tissue-specific expression for selected ceRNA interactions: CDR1as and miR-7, and CircSPHKAP and miR-1. R package ggplot2 v3.3.2 (https://cran.r-project.org/web/packages/ggplot2/index.html) was used to create the figure.

We employed Pareto Frontier Analysis to integrate circRNA-miRNA binding data with their expression data, aiming to improve functional relevance of the predicted ceRNA interactions. Figure 2 gives 2531 circRNA-miRNA interaction pairs on the first 30 Pareto fronts with the top combination scores due to either strongest circRNA-miRNA binding potentials and/or highest co-expression from ENCODE. Nine circRNA-miRNA interactions, including eight unique circRNAs and five unique miRNAs, are located on the first Pareto front. Among those is the well-studied CDR1as-miR7 interaction, where 74 miR7 binding sites were predicted over the entire body of CDR1as. This circRNA-miRNA pair was also found to be co-expressed in several tissues, such as adrenal gland (circRNA SRPBM = 1680.4; miRNA CPM = 1032.4) and thyroid gland (circRNA SRPBM = 1843.5; miRNA CPM = 3503.0).

Besides the abovementioned interactions with both strong co-expression and binding scores, the Pareto method also highlights circRNA-miRNA pairs with unequal interaction strengths from two data sources. CircSPHKAP and miR-1-3p is one such example that ranks among the top Pareto fronts (front 19; ${S}_{P}^{\text{circ}|\text{mir}}$= 0.9993254) due to strong evidence from circRNA-miRNA co-expression (${S}_{\text{exp}}^{\text{circ}|\text{mir}}$= 0.9938584) and relatively weaker binding potential (0.27 MREs/Kb). Further scrutinization revealed that circSPHKAP (chr2: 228,881,121–228,884,872) was exclusively expressed in heart tissues (circRNA SRPBM = 1055.9, rank: 62/9281 in hearts tissues) with no detectable back-splicing junction counts in the rest of ten tissues from ENCODE, which is consistent with findings from a recent study supporting the use of circSPHKAP as a biomarker for cardiomyocytes^95^. On the other hand, miR-1-3p was among the highly expressed miRNA in heart tissues (CPM = 212,747), which was also known to be directly involved or implicated in various heart and cardiovascular diseases, including hypertrophic cardiomyopathy, coronary artery disease, myocardial infarction, heart failure and stroke^96^. Interestingly, circSPHKAP was reported to have dynamic expression changes in human induced pluripotent stem cell derived cardiomyocytes during cardiac development^97^, which further underscores its potential functional role in cardiac tissues.

**Figure 3:**
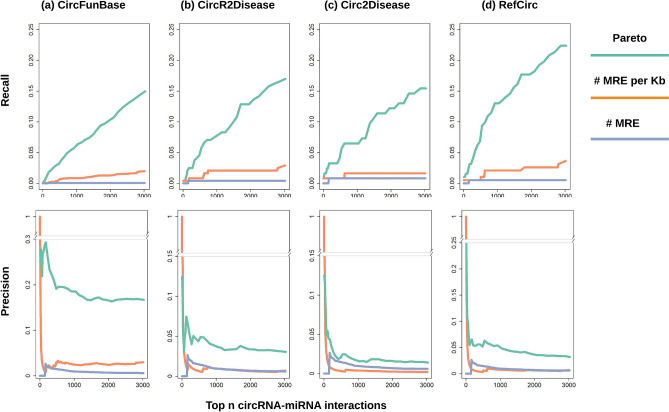
Evaluating the performance of different circRNA-miRNA interaction ranking methods using data from several circRNA function/disease databases. Upper panel: recall (sensitivity) curve. Lower panel: precision. R package plotrix v3.7–8 (https://cran.r-project.org/web/packages/plotrix/index.html) was used to create gap plot.

To systematically evaluate the advantage of integrating circRNA-miRNA co-expression with MRE data over conventional MRE-based approaches, we performed functional relevance analysis of the top ranked circRNAs. We used circRNAs from CircFunBase^49^ that were previously reported to be differentially expressed in one or more disease studies as one of the references. Figure 3a upper panel gives the percentages of overlap between circRNAs from the top-n ($1\le n\le 3000$) circRNA-miRNA interactions and those from CircFunBase based on three different ranking methods: the Pareto method, total number of MREs (nMRE), length-normalized number of MRE (i.e., number of MREs per kilo bases: nMRE/Kb). Apparently, Pareto integration of co-expression data with binding data significantly improved the recall of known circRNAs from CircFunBase: ~ 15% from the Pareto method compared to 2% from the length-normalized MRE method. It is worth noting that using the total number MREs yielded very low recall compared to its length-normalized counterpart. Moreover, the precision of the Pareto method was also significantly increased (Fig. 3a lower panel). Additionally, we applied similar analysis on three more circRNA databases, including CircR2Disease^98^, Circ2Disease^99^, and RefCirc (http://www.ncvar.org/RefCirc/index.php), which contained annotated disease-associated circRNAs from independent research groups. Figure 3b–d show that circRNAs prioritized by the Pareto method was consistently more enriched in known disease associations by several folds, in terms of both precision and recall, which provided strong evidence that incorporation of co-expression data significantly increased functional relevance of prioritized ceRNA interactions.

Moreover, we further validated our Pareto-ranked miRNA-gene interactions on three additional miRNA target databases: miRDB^100,101^, miRTAR^102^, and miRWalk^103^. We considered the top 3000 miRNA-gene interactions ranked by Pareto, TargetScan, and miRTarBase and found that Pareto improved the performance, in terms of precision and recall, on all three databases (Supplementary Fig. S3). This demonstrates Pareto’s utility in combining multiple pieces of information, namely TargetScan and miRTarBase, to improve overall performance.

### Cerina shiny server interface

We developed a user-friendly R Shiny web application of Cerina for researchers to visualize ceRNA interactions and perform circRNA functional enrichment analysis. The tool consists of three main components: data exploration, miRNA-circRNA network visualization, and functional enrichment analysis.

**Figure 4:**
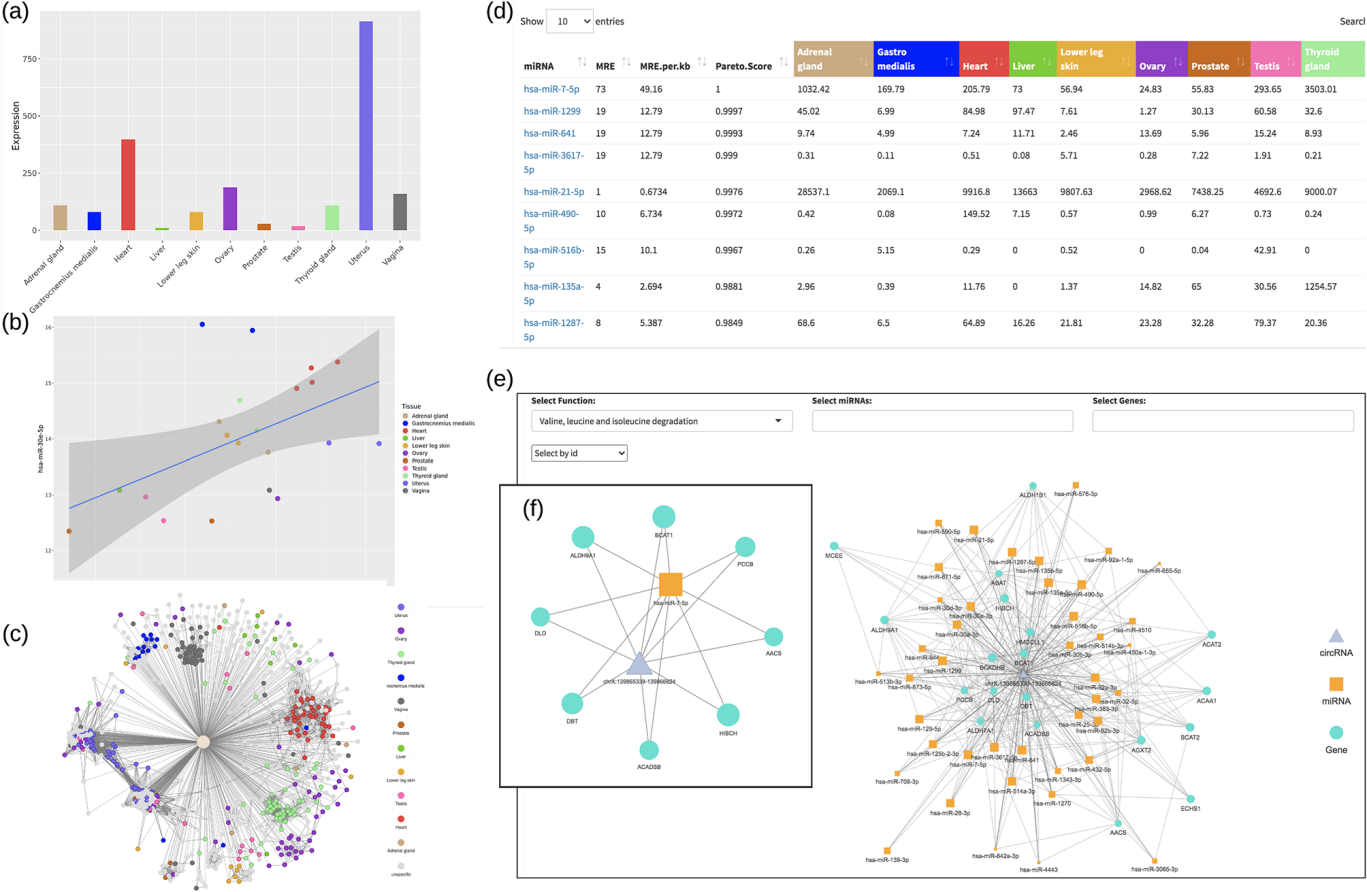
Web interface of Cerina. (**a**,**b**) In the data exploration section, users can query an mRNA, miRNA, or circRNA to examine its expression across different tissues. Users can also perform correlation analysis for ceRNA interactions. (**c**) MiRNA-circRNA interaction page allows users to visualize and report tissue-specific miRNA-circRNA interactions as a network. (**d**) CircRNA-miRNA exploration tab reports all miRNAs interacting with a selected circRNA as a downloadable table displaying MRE, Pareto score, and miRNA expression. (**e**) Functional analysis tab reports significantly enriched pathways and gene ontology terms that can be visualized as a network displaying the selected circRNA with interacting miRNAs and genes. (**f**) Users can choose a subset of miRNAs/genes/functions to display in the network. R packages ggplot2 v3.3.2 (https://cran.r-project.org/web/packages/ggplot2/index.html), visNetwork v2.0.9 (https://cran.r-project.org/web/packages/visNetwork/index.html), and DT v0.15 (https://cran.r-project.org/web/packages/DT/index.html), were used to create the figure.

In the data exploration section (Fig. 4a,b), users can query individual circRNAs, miRNAs, and linear RNAs to view their expression profiles across the 11 ENCODE tissues. Additionally, correlation analysis of any queried pair can be visualized via scatterplots. The miRNA-circRNA network page allows users to query an individual miRNA to visualize a network of its interacting circRNAs (Fig. 4c). A downloadable table listing all circRNAs plotted in the network is also provided. The table shows detailed information such as parental gene, tissue specific expression, number of MREs, number of MREs per kilobase, and the Pareto score. Finally, in the functional enrichment component, users can enter an individual circRNA to visualize a network and download a table including all interacting miRNAs (Fig. 4d). Here, users have the option to run the permutation test using either all interacting miRNAs or a subset of “top” miRNAs to identify target genes. After running the permutation test, users can proceed to functional enrichment analysis of significant genes (e.g., p-value ≤ 0.05) on KEGG^69–72^ pathways or gene ontology (GO)^72–74^ terms. The enrichment results of a circRNA are output as a downloadable table that can be categorized by either the functional term (i.e., KEGG or GO term) or the binding miRNA, with graphic visualization also made available (Fig. 4e). Cerina allows users to choose a subset of miRNA/genes/functions to display in the graph (Fig. 4f). A detailed tutorial is accessible at the Cerina website.

### Case studies on differentially expressed circRNAs

#### ceRNAs in cancers: integrative analysis with TCGA data

We first performed Cerina analysis on gastric cancer^104^ and prostate cancer^65^ datasets to demonstrate its utility to identify potential roles of circRNAs in functional sequestration of miRNAs. Enrichment analysis validated that Cerina-predicted miRNAs and their target genes were strongly associated with gastric and prostate cancer annotated by Human MicroRNA Disease Database (HMDDv3.2)^96^ and KEGG (Supplementary Table S2). To further explore the potential roles of circRNAs as competing endogenous RNAs in tumorigenesis, we further analyzed miRNA and mRNA expression data from The Cancer Genome Atlas^105,106^, allowing construction of ceRNA networks with expression changes of circRNA/mRNA that were inversely correlated with those of the miRNAs (Supplementary Methods).

In the gastric cancer dataset, circARHGEF12 (hsa_circ_0002089; chr11: 120,347,369–120,348,235), a circRNA that was down-regulated in cancer, was significantly enriched in both miRNA and pathway enrichment analysis (Supplementary Tables S2, S3). Two precursor miRNAs, hsa-mir-134 and hsa-mir-590, with predicted MREs on circARHGEF12 were significantly up-regulated in TCGA gastric cancer dataset. Differential gene expression analysis of TCGA mRNA-Seq data identified 12 genes from the KEGG gastric cancer pathway that were down-regulated in cancer tissue, eight of which had predicted interactions in Cerina with the two up-regulated miRNAs. SMAD4, hub of TGFβ signaling and a tumor suppressor for gastrointestinal carcinogenesis^107^ was among the down-regulated target genes of circARHGEF12, suggesting a potential tumorigenesis effect caused by un-sequester of oncogenic miR-134 and miR-590^108,109^. Catenin Alpha (CTNNA1 and CTNNA2) expression was also down-regulated in cancerous tissues, which was consistent to well-reported tumor-suppressor functions of CTNNA1 and CTNNA2 in various cancers^110–112^. Interestingly, CTNNA2 was previously predicted to be part of lincRNA-mediated miR-590-3p sponge network (http://cis.hku.hk/GastricCancerMAP/index.php), unveiling a novel role of circARHGEF12 in gastric carcinogenesis and its involvement in complicated wiring of ceRNA interactions harboring miR-590.

**Figure 5:**
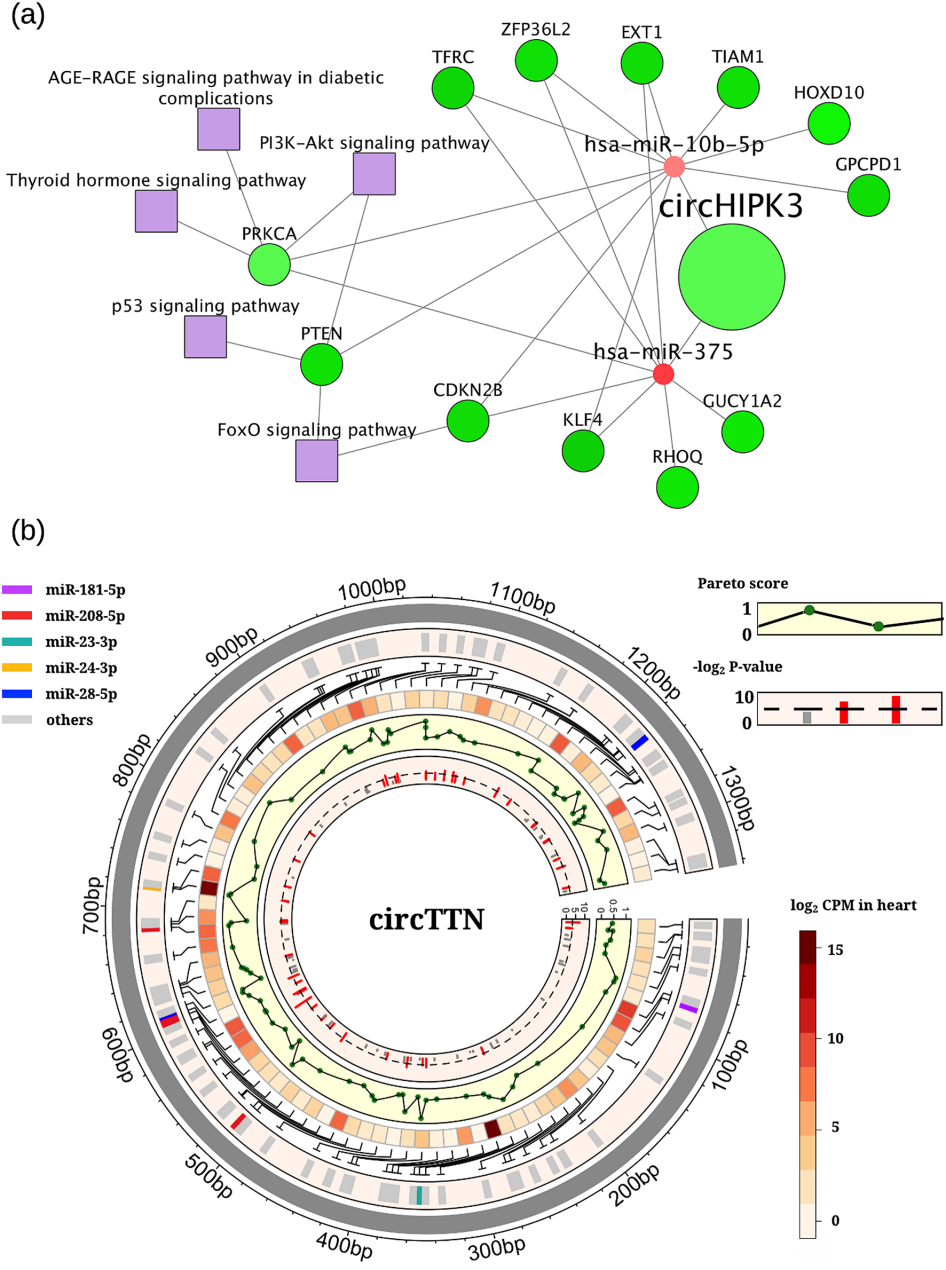
Cerina links circRNAs with potential functions in cancer and cardiovascular disease. (**a**) Network view of predicted circRNA-miRNA-gene-function associations for circHIPK3 in prostate cancer. For circRNA, miRNA, and genes, their colors represent their fold changes (red: up-regulated in prostate cancer; green: down-regulated in prostate cancer). (**b**) Circos plot showing predicted miRNAs of circTTN with Pareto score, expression in heart tissues (log_2_ CPM), and enrichment in immunology functions (-log_2_ p-value). Cytoscape v3.5.1 was used to create the circRNA-miRNA-gene-function network figure (https://cytoscape.org/). R package circlize v0.4.8 (https://cran.r-project.org/web/packages/circlize/index.html) was used to make circos plot.

In the prostate cancer study comparing localized primary prostate adenocarcinoma and matched normal tissues, five differentially expressed circRNAs: circHIPK3 (hsa_circ_0000284; chr11: 33,307,958–33,309,057), circN4BP2L2 (hsa_circ_0000471; chr13: 33,091,993–33,101,669), circUNC13B (hsa_circ_0008518; chr9: 35,295,692–35,313,986), circZCCHC6 (hsa_circ_0001869; chr9: 88,920,106–88,924,932), and circSENP6 (hsa_circ_0001614; chr6: 76,412,360–76,412,788), had significant enrichment in miRNAs related to “carcinoma, prostate” and KEGG “prostate cancer” pathway (Supplementary Tables S2, S4). CircHIPK3, in particular, was one of the most abundantly expressed circRNA with an average log expression (SRPBM) of 12.1, compared to the median average log expression of 2.4 among all detected circRNAs in prostate tissues. Dysregulation of circHIPK3 was frequently reported in multiple cancers^113^. Interestingly, both up- and down-regulation of circHIPK3 were identified in tumor tissues, indicating a dual role of circHIPK3 in cancer to regulate tumor progression through sponging different miRNAs^114^. As a side note, the host gene of circHIPK3 was also significantly down-regulated in the prostate cancer tissues, which was further confirmed by an independent TCGA RNA-Seq data. Integrative analysis of TCGA RNA-Seq/miRNA-Seq data revealed that two Cerina-predicted miRNAs mir-10b and mir-375 that can be sequestrated by circHIPK3 were up-regulated in TCGA prostate cancer samples. Oncogenic functions of mir-10b has been well-documented in various cancers, including oral cancer^115^, head and neck cancer^116^, hepatocellular carcinoma^117^, breast cancer^118^, and colon cancer^119^. Top mir-10b targets (Pareto score ${S}_{P}^{\text{mir}|\text{gene}}$ ≥ 0.95) that were also down-regulated in tumors included HOXD10, KLF4, and PTEN, all of which had well-known tumor-suppressor functions and reduced expression in prostate cancer tissues^120–122^. It is worth noting that depletion of mir-10b restored PTEN expression in breast cancer, which led to decreased cancer stem cell renewal through inhibition of AKT^118^. Mir-375, another highly expressed miRNA that can be sponged by circHIPK3, was well described as a tumor suppressor in many cancers, yet its expression was found to be up-regulated in breast and prostate cancers^123^. Consistently, our analysis of TCGA prostate miRNA-Seq data showed significant up-regulation of mir-375 (log2FC = 1.874, adjusted p-value = 4.85E−43). Top down-regulated mir-375 target genes (${S}_{P}^{\text{mir}|\text{gene}}$≥ 0.95) included tumor suppressors such as ZFP36L2, CDKN2B, PRKCA, KLF4, and EXT1, suggesting possible protumorigenic activity of circHIPK3 implemented through ceRNA interaction with mir-375. Figure 5a gives the circHIPK3-centered ceRNA interaction network, connecting mir-10 and mir-375 to significant dysregulated KEGG pathways, including PI3K-AKT signaling and p53 signaling pathways.

#### Reduced circTTN expression correlates with down-regulation of immune response in end-stage dilated cardiomyopathy

In the dilated cardiomyopathy (DCM) dataset^124^, circTTN (hsa_circ_0141774; chr2: 179,542,851–179,585,929) had the highest abundance among all differentially expressed circRNAs. circTTN was also exclusively expressed in heart tissues (Supplementary Fig. S4). Cerina analysis revealed that circTTN had binding sites for 82 miRNAs, among them seven mature miRNAs (miR-23b-3p, miR-23a-3p, miR-24-3p, miR-181a-5p, miR-28-5p, miR-181b-5p, miR-208a-5p) with Pareto scores greater than 0.95 (Fig. 5b). Functional enrichment analysis showed that targets of these seven miRNAs were highly associated with cardiovascular and circulatory system development, which were also significantly enriched in several pathways, including TNF, FoxO, and ERBB signaling pathways. In an independent gene expression study (GSE3586)^125^, Barth et al. reported that end-stage DCM was characterized by excessive down-regulation of “immune response”, “inflammatory response”, and “chemokine activity”. Given the apparent under-expression of circTTN in DCM, we hence seek to investigate the possibility of circTTN-mediated suppression of immunity and inflammatory response through ceRNA interactions. Consistently, over-expression of mir-23a, mir-24, mir-28, and mir-208 in DCM patients were previously reported in one or more studies^126–129^, which suggested a direct link between increased miRNA activity and reduced circTTN expression levels in DCM. Moreover, enrichment analysis showed that target genes of mir-23a/b were significantly associated with the down-regulation of immune-related genes in Barth’s study (Supplementary Methods), exampled by binding of mir-23a/b to CCL2, a chemokine with the most significant down-regulation in DCM. Taken together, deregulation of circTTN correlates with down-regulation of immune response in end-stage DCM patient likely through modulation of mir-23a/b and others.

## Conclusion and discussion

Cerina is the first systematic circRNA functional annotation tool based on integrative analysis of competing endogenous RNA interactions. It has a collection of more than 1.5 million inferred ceRNA interactions between over 33,000 circRNAs and hundreds of miRNAs detected in 11 ENCODE tissues. Although many databases were established for searching MREs in circRNAs, none of them incorporated gene expression data for predicting circRNA-miRNA interactions. Guo et al., raised concerns regarding the functional role of thousands of low-expressing circRNAs, strongly suggesting that the expression levels of circRNAs to be taken into account when interpreting circRNA functions^66^. On a similar note, TargetScan also argued that, in order to mediate consequential repression of its targets, the expression of a miRNA should reach an adequate level, hence recommending removing false-positive interactions based on miRNA expressions levels (http://www.targetscan.org/vert_72/docs/FP_noncons.html). By integrating paired circRNA and miRNA expression data with the predicted MREs using a Pareto Frontier Analysis framework, we have significantly improved the accuracy and functional relevance of the identified ceRNA interactions, validated by data from several mainstream circRNA-disease databases. Through Cerina’s Shiny web interface, users can perform functional query of a circRNA to retrieve information regarding its most likely sponged miRNAs and their tissue-specific expressions, down-stream target genes, and potential enriched biological functions and pathways.

Although applicable to various disease studies, one major limitation of Cerina is that its entire functional prediction is built upon the circRNA-miRNA-mRNA axis. While this miRNA-sponge paradigm is under the spotlight for human circRNA research^18–39^, increasing evidences have supported a number of alternative biological mechanisms, including alternative splicing regulation, RNA-binding protein sponge, posttranscriptional gene regulation, and protein-coding, among others^3,7–13^.

In the context of human cancer, circCcnb1 may directly bind H2ax and wild-type p53, which attenuates tumor-suppressor function of p53 and promotes cell proliferation by allowing Bcl2-Bclaf1 binding. On the contrary, in p53 mutant cells, the circCcnb1-H2ax compound binds Bclaf1, hence activates Bclaf1 tumor-suppressor function and leads to apoptosis^130^. In another breast cancer study, co-localization of circ-Amotl1 and c-myc was detected, suggesting abnormal levels of circAmotl1 to facilitates c-myc nuclear translocation through direct circRNA-protein binding^131^. In human glioma, a new protein encoded by circFBXW7 was discovered to have inhibitory effect of cell cycle and proliferation^132^.

Similarly, in non-cancer diseases, various “non-sponging” mechanistic models have been proposed for circRNAs in disease pathogenesis and progression. Examples include circ-Foxo3, a circRNA that promotes cardiac senescence binds CDK2 and p21 to form a ternary complex, blocking cell cycle progression^133^. Also, in a systemic lupus erythematosus (SLE), degraded circRNAs upon viral induction in monocytes was found to form short RNA duplexes that inhibited abnormal protein kinase R activation cascade, highlighting a new role of circRNA in autoimmune diseases due to its unique structure^134^.

On the other hand, due to the complex ceRNA networking in mammalian cells, circRNAs are a potent family, yet not the only one, of being capable of regulating protein-coding genes by sequestration of miRNAs. Other than circRNAs, small non-coding RNAs, pseudogenes, and lincRNAs, all actively participate in ceRNA interaction network through competing of shared miRNAs^14,135–137^. Therefore, when Cerina predicts strong ceRNA associations that lead to functional outcomes, experimental validations are needed to further confirm the identified interactions, such as use pull-down assay and dual-luciferase reporter assay to confirm circRNA-miRNA binding^31,34,138^, or over-express/silence circRNA or their interacting miRNAs to further verify predicted ceRNA interactions and associated phenotypic changes^31,34,139,140^.

## Supplementary Information

Supplementary Information.

Supplementary Table S1.

Supplementary Table S2.

Supplementary Table S3.

Supplementary Table S4.

## Data Availability

A web service of Cerina can be accessed through: https://www.bswhealth.med/research/Pages/biostat-software.aspx.

## Code availability

Source code for Cerina is available through GitHub at https://github.com/jcardenas14/CERINA.
