# Supplementary material for: Cerina: systematic circRNA functional annotation based on integrative analysis of ceRNA interactions
Source: Sci Rep. 2020 Dec 17;10:22165. doi: 10.1038/s41598-020-78469-x (PMC7746713; doi:10.1038/s41598-020-78469-x)
Supplement: Supplementary file 1 — Supplementary Information. [file 41598_2020_78469_MOESM1_ESM.pdf]

Supplementary

Cerina: systematic circRNA functional annotation based on integrative analysis of ceRNA interactions

Jacob Cardenas[1,†], Uthra Balaji[1,†], and Jinghua Gu[1,*]

[1]Baylor Scott & White Research Institute, Dallas, TX 75204, USA

* To whom correspondence should be addressed. † Joint Authors. Tel: 1(214) 820-9912; Email: Jinghua.Gu@BSWHealth.org

Supplementary Methods 2
Combining multiple algorithms for circRNA detection 2
A hierarchical framework to predict mature sequences for novel circRNAs 2
2-D illustration of Pareto Frontier Analysis........ 2
MiRNA and pathway level disease enrichment analysis........ 3
Additional information for data analysis in case studies 3
a) TCGA gastric and colorectal cancer miRNA-Seq and RNA-Seq data analysis..... 3
b) Dilated cardiomyopathy gene expression data analysis 4
Supplementary Figures 5
Supplementary Tables........ 7
References........ 7

Supplementary Methods

Combining multiple algorithms for circRNA detection

Figure S1 gives the schematic of the combination framework. First, CIRI2 (BWA index built on genome version hg19) was performed on total RNA-Seq data from 11 organs and identified 34,323 back-splicing (BS) junctions with at least 3 supporting BS reads. For CIRI2-detected BS junctions with 2 or fewer reads, we checked if they were also detected by an independent algorithm (i.e., CIRCexplorer). Those CIRI2 BS junctions that were neither confirmed by CIRCexplorer nor collected by circAtlas [1] were considered as false positives and were not included in downstream analysis. Finally, a total of 33,461 BS junctions were reported from 11 ENCODE tissues (Supplementary Table S1). CircRNA counts were normalized using the size factors calculated from total RNA-Seq data. After MRE prediction (described in next section), six circRNAs without predicted miRNA binding sites were further removed, yielding a total of 33,455 circRNAs into the final analysis.

A hierarchical framework to predict mature sequences for novel circRNAs

Among a total of 34,323 circRNAs from ENCODE [2] tissues, mature sequences for 30,282 circRNAs were extracted from circAtlas (hg19) v1909. Of the remaining 4,041 circRNAs, 242 circRNAs had predicted mature sequence available from circBase [3]. An in-house script was used to predict the mature sequence for the remaining 3799 'novel' circRNAs. Briefly, GENCODE (v19) annotation was used to extract exons of transcripts that overlap each novel circRNA. First, all transcripts that overlap with a given circRNA were identified. For circRNAs with more than one overlapping transcript, the transcript with highest coverage was used to predict the final mature circRNA sequence. When circRNAs were not covered by GENCODE annotation, lincRNA annotation obtained from UCSC (hg19) was used. Finally, for circRNAs with no annotation from either GENCODE or UCSC lincRNA, genomic sequences were used as mature sequences.

2-D illustration of Pareto Frontier Analysis

Figure S2 gives a brief workflow to rank 2D scores using Pareto Frontier Analysis. (a-b) In the initial round, all data points were evaluated and those were not Pareto-dominated by any other data points were selected as the first Pareto frontier. (c) Once the first Pareto frontier is determined, it will be "peeled off" from the whole data set. (d) Based on the remaining data points, the second Pareto frontier will be formed. Points on the second font are non-dominated by the rest of the data. (e-g) Such procedure will be repeatedly performed until all data points were assigned to a certain Pareto frontier. The above method will generate a series of ordered Pareto frontiers with varying sizes, thus achieving ranking of 2D data. Points on the same Pareto frontier have equal ranks.

MiRNA and pathway level disease enrichment analysis

To evaluate whether Cerina can effectively retrieve relevant information regarding the functions of the differentially expressed circRNAs, miRNA and pathway enrichment analysis were performed. In the miRNA enrichment analysis, for any individual circRNA from a given disease study, we evaluated the overlap of top miRNAs prioritized by Cerina with the curated miRNA set for the corresponding diseases from HMDDv3.2. Fisher's exact test was used to calculate the statistical significance of the overlap. For example, the predicted miRNAs (circRNA-miRNA Pareto score ≥ 0.95) for 12 out 18 (67%) circRNAs from the gastric cancer study were significantly (Fisher's exact test p-value ≤0.05) associated with "carcinoma, gastric", strongly suggesting the potential role of Cerina-prioritized circRNA-miRNA interactions in gastric cancer pathogenesis (Supplementary Table S2). Further "meta" p-value analysis using Fisher's combination method [4] gives an overall enrichment p-value of 4.76E-12 for all circRNAs from the gastric cancer dataset. Such significant disease associations were also observed in the prostate cancer dataset (54/98 of circRNA had p-value ≤0.05; meta p-value = 6.88E-63).

In a similar vein, we also evaluated whether genes that were predicted to interact with a given circRNA through ceRNA networks were significantly associated with known pathways of that specific disease. To this end, genes with significant ceRNA interactions (p-value ≤ 0.01) with an individual queried circRNA were extracted to performed overlap analysis with Kyoto Encyclopedia of Genes and Genomes (KEGG) [5] pathways using Fisher's exact test. Pathway enrichment p-values for all circRNAs within the same study were further aggregated to calculate the combined "meta" p-value using Fisher's p-value combination method. For instance, 13 out of the 98 circRNAs from the prostate cancer study were significantly enriched (p-value ≤0.05) in KEGG pathway: "prostate cancer" with a combined p-value of 8.04E-10, which indicated that the differentially expressed circRNAs might collectively sequester miRNAs responsible for prostate cancer pathogenesis.

Additional information for data analysis in case studies

a) TCGA gastric and colorectal cancer miRNA-Seq and RNA-Seq data analysis

TCGA RNA-Seq and miRNA-Seq data for gastric [6] and prostate cancer [7] were retrieved from the Genomic Data Commons (GDC) portal [8]. Differential gene/miRNA expression analysis comparing cancer versus normal was performed with DESeq2 [9]. Genes and miRNAs were considered differentially expressed if they survived a false discovery rate (FDR) ≤ .05.

Consistent with the methods described previously, we integrated TCGA gene/miRNA expression data with circRNA data using the following approach. First, we performed circRNA functional enrichment analysis on circRNAs reported as differentially expressed in gastric/prostate cancer data sets and only considered those that were also significantly enriched (p-value ≤ 0.05) in their corresponding KEGG pathway (i.e., KEGG "gastric cancer" and "prostate cancer"). Then, for each of these circRNAs, we performed miRNA set analysis using miRNAs with Pareto score ≥ 0.95. Fisher's exact test was used to test for over-representation of an input miRNA list with the Human microRNA Disease Database (HMDD) [10] collection of disease related miRNA sets. We only considered

circRNAs whose interacting miRNAs were significantly enriched (p-value ≤ 0.05) in their respective diseases. For gastric cancer, one down-regulated circRNA was considered: chr11:120347369-120348235. For prostate cancer, two up-regulated (chr9:35295692-35313986, chr9:88920106-88924932) and three down-regulated (chr6:76412360-76412788, chr11:33307958-33309057, chr13:33091993-33101669) circRNAs were considered. Finally, we filtered the list of interacting miRNAs and their target genes by only considering those that were differentially expressed in opposite directions. In other words, for an up-regulated circRNA, we selected down-regulated miRNAs and their up-regulated target genes, and vice versa for down-regulated circRNAs. MicroRNAs with a base mean smaller than 20 normalized counts were excluded from the analysis. Supplementary Tables S3-S4 summarizes these results.

b) Dilated cardiomyopathy gene expression data analysis

Microarray gene expression data from GSE3586 that contained 13 DCM and 15 non-failing hearts (NF) were analyzed by GEO2R (https://www.ncbi.nlm.nih.gov/geo/query/acc.cgi?acc=GSE3586). Using an adjusted p-value threshold of 0.05, 3986 probes were identified to be significantly down-regulated. We also extracted genes annotated as “immune response” (GO0006955), “inflammatory response” (GO0006954), and “chemokine activity” (GO0008009) from the Gene Ontology Resources (http://geneontology.org) to compile a list of 2631 “immune-related genes”. We take the intersection of the genes annotated to the 3986 probes (multiple mapping removed) and the immune-related genes to derive the final a list of “down-regulated immune-related genes” in DCM. Chi-squared test is used to test the association of targets of circTTN-interacting miRNAs and immune down-regulation.

Supplementary Figures

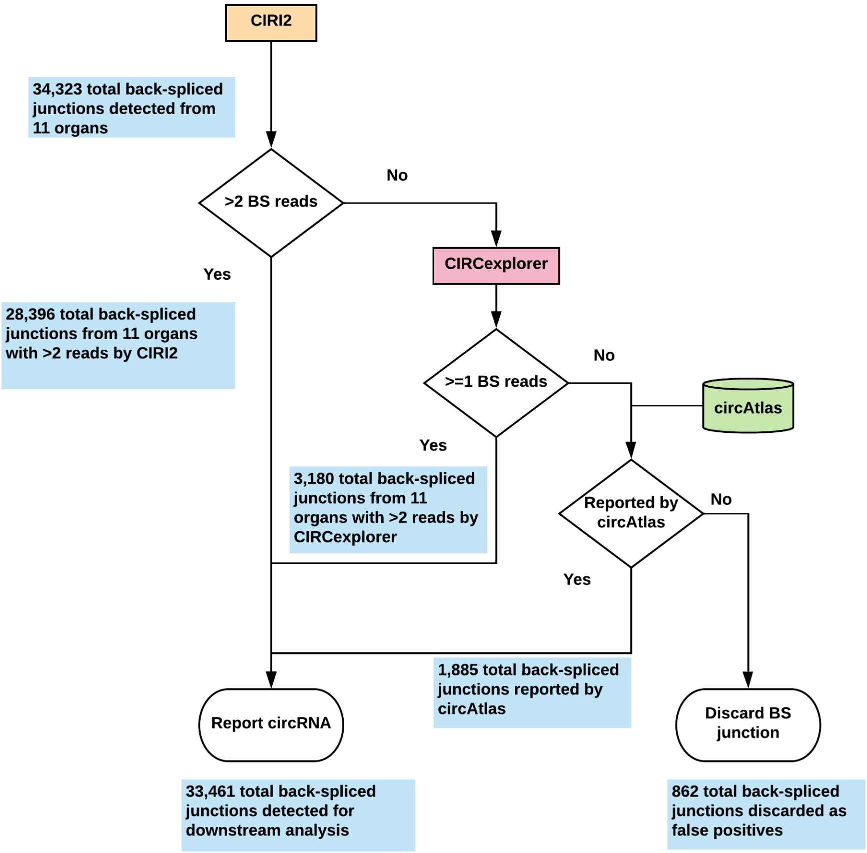

Figure S1 Combine two circRNA detection algorithms to reduce false positive detections.

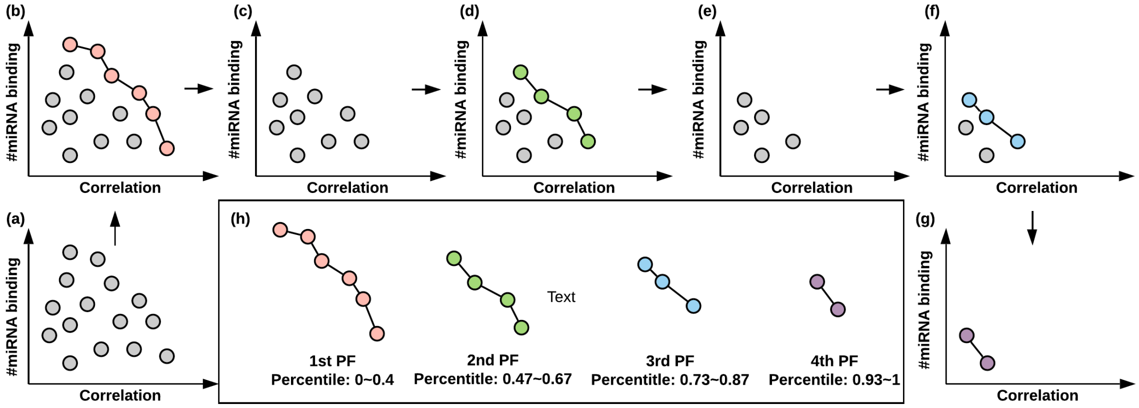

Figure S2 Illustration of Pareto Frontier Analysis on a 2D case. (a-b) Formation of the first frontier; (c-d) Formation of the second frontier; (e-f) Formation of the third frontier; (g) Formation of the fourth (last) frontier; (h) Points on each frontier are associated with different percentiles.

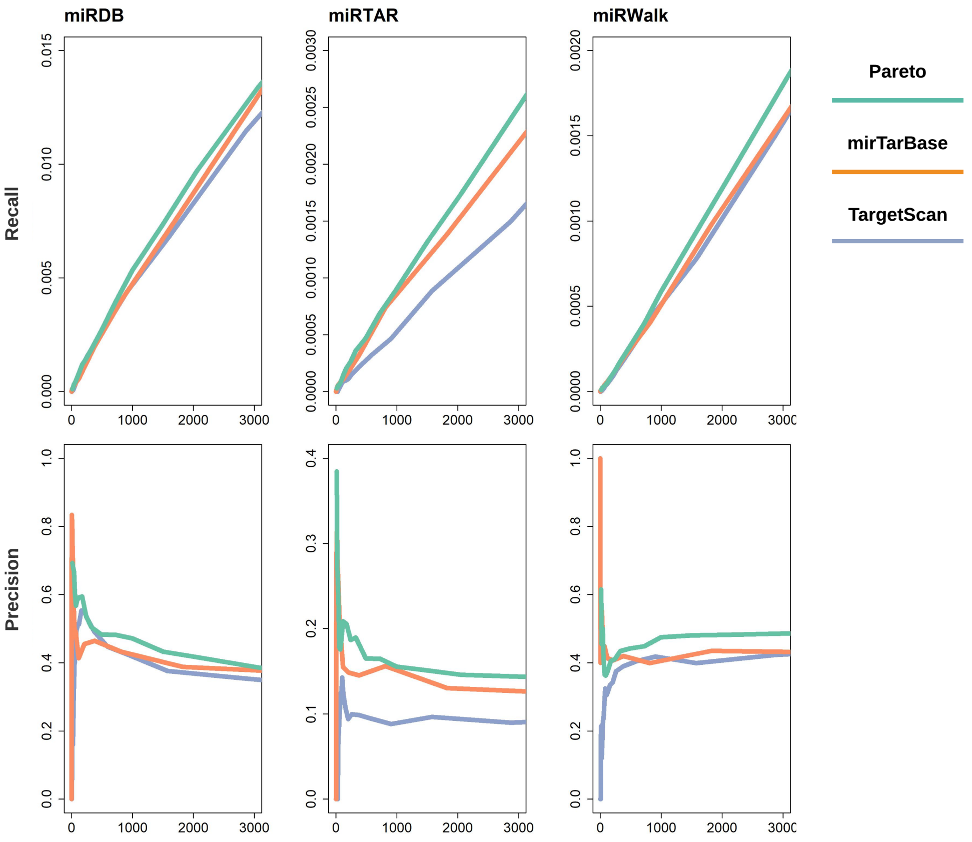

Figure S3 Precision and recall for comparing miRNA-gene interaction predictions using three public miRNA target databases.

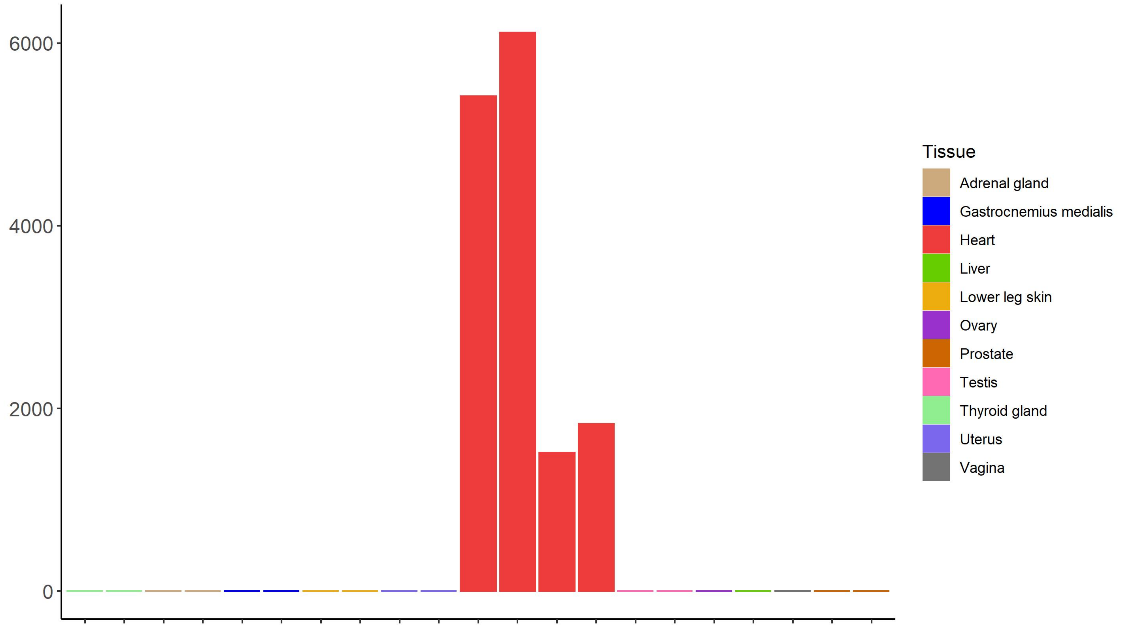

Figure S4 CircTTN is exclusively expressed in ENCODE heart tissues.

Supplementary Tables

The following supplementary tables are provided in separate spreadsheets.

Table S1 List of tissue samples from ENCODE.
See “Supplementary Table S1 List of tissue samples from ENCODE.xlsx”.

Table S2 Disease enrichment analyses at miRNA-set and pathway levels for gastric cancer and prostate cancer.
See “Supplementary Table S2 Disease enrichment analysis at miRNA-set and pathway levels for gastric and prostate cancer.xlsx”.

Table S3 CircRNAs, miRNAs, and target genes from gastric cancer integrative analysis.
See “Supplementary Table S3 Gastric cancer integrative analysis.xlsx”.

Table S4 CircRNAs, miRNAs, and target genes from prostate cancer integrative analysis.
See “Supplementary Table S4 Prostate cancer integrative analysis.xlsx”.

References

1 Ji, P. *et al.* Expanded Expression Landscape and Prioritization of Circular RNAs in Mammals. *Cell Rep* **26**, 3444-3460 e3445, doi:10.1016/j.celrep.2019.02.078 (2019).
2 Davis, C. A. *et al.* The Encyclopedia of DNA elements (ENCODE): data portal update. *Nucleic Acids Res* **46**, D794-D801, doi:10.1093/nar/gkx1081 (2018).
3 Glazar, P., Papavasileiou, P. & Rajewsky, N. circBase: a database for circular RNAs. *RNA* **20**, 1666-1670, doi:10.1261/rna.043687.113 (2014).
4 Fisher, R. A. *Statistical methods for research workers.*, (Edinburgh, Oliver and Boyd., 1925).
5 Kanehisa, M. & Goto, S. KEGG: kyoto encyclopedia of genes and genomes. *Nucleic Acids Res* **28**, 27-30, doi:10.1093/nar/28.1.27 (2000).
6 Cancer Genome Atlas Research, N. Comprehensive molecular characterization of gastric adenocarcinoma. *Nature* **513**, 202-209, doi:10.1038/nature13480 (2014).
7 Cancer Genome Atlas Research, N. The Molecular Taxonomy of Primary Prostate Cancer. *Cell* **163**, 1011-1025, doi:10.1016/j.cell.2015.10.025 (2015).
8 Grossman, R. L. *et al.* Toward a Shared Vision for Cancer Genomic Data. *N Engl J Med* **375**, 1109-1112, doi:10.1056/NEJMp1607591 (2016).
9 Love, M. I., Huber, W. & Anders, S. Moderated estimation of fold change and dispersion for RNA-seq data with DESeq2. *Genome Biol* **15**, 550, doi:10.1186/s13059-014-0550-8 (2014).
10 Huang, Z. *et al.* HMDD v3.0: a database for experimentally supported human microRNA-disease associations. *Nucleic Acids Res* **47**, D1013-D1017, doi:10.1093/nar/gky1010 (2019).
